# Phospholipase D1 regulation of TNF-alpha protects against responses to LPS

**DOI:** 10.1038/s41598-018-28331-y

**Published:** 2018-07-03

**Authors:** Marc-Andre Urbahn, Sonja Charlotte Kaup, Friedrich Reusswig, Irena Krüger, Martina Spelleken, Kerstin Jurk, Meike Klier, Philipp A. Lang, Margitta Elvers

**Affiliations:** 1 Department of Vascular and Endovascular Surgery, Heinrich-Heine-University University Medical Center, Moorenstraße.5, 40225 Düsseldorf, Germany; 2 Center for Thrombosis and Hemostasis (CTH), University Medical Center Mainz, Mainz, Germany; 3 Department of Molecular Medicine II, Heinrich Heine University, Düsseldorf, Germany

## Abstract

Sepsis is a systemic inflammatory disorder with organ dysfunction and represents the leading cause of mortality in non-coronary intensive care units. A key player in septic shock is Tumor Necrosis Factor-alpha (TNF-α). Phospholipase (PL)D1 is involved in the regulation of TNF-α upon ischemia/reperfusion injury in mice. In this study we analyzed the impact of PLD1 in the regulation of TNF-α, inflammation and organ damage in experimental sepsis. PLD1 deficiency increased survival of mice and decreased vital organ damage after LPS injections. Decreased TNF-α plasma levels and reduced migration of leukocytes and platelets into lungs was associated with reduced apoptosis in lung and liver tissue of PLD1 deficient mice. PLD1 deficient platelets contribute to preserved outcome after LPS-induced sepsis because platelets exhibit an integrin activation defect suggesting reduced platelet activation in PLD1 deficient mice. Furthermore, reduced thrombin generation of PLD1 deficient platelets might be responsible for reduced fibrin formation in lungs suggesting reduced disseminated intravascular coagulation (DIC). The analysis of *Pld1*^*fl/fl*^-PF4-Cre mice revealed that migration of neutrophils and cell apoptosis in septic animals is not due to platelet-mediated processes. The present study has identified PLD1 as a regulator of innate immunity that may be a new target to modulate sepsis.

## Introduction

Sepsis is a systemic inflammatory disorder resulting from infection of invading microorganisms and represents the leading cause of mortality in non-coronary intensive care units worldwide^1^. Patients suffer from fever, tachycardia and tachypnea in association with local or systemic infection. Severe sepsis is defined as sepsis with organ dysfunction including hypotension, hypoxemia, oliguria, metabolic acidosis, etc. Microorganisms are believed to initiate these symptoms either by direct invasion of the host’s blood stream, by the elaboration of exotoxins or by both^2^. Moreover, sepsis is associated with hemostatic abnormalities such as activation of blood coagulation (hypercoagulability) that contributes to localized venous thrombosis, to acute disseminated intravascular coagulation (DIC) with massive thrombin formation and microvascular thrombosis responsible for the multiple organ dysfunction syndrome - at least in part - with consumption of platelets and coagulation proteins^1^.

Phospholipid mediators play an important role in inflammatory processes. They were produced upon inflammation and trigger different signaling cascades mostly via G-protein-coupled receptors to modulate cell adhesion to the vessel wall, infiltration of leukocytes and chemokine release^3^. Phospholipase (PL)D catalyzes the hydrolysis of phosphatidylcholine into phosphatidic acid (PA) and choline^4^. PA as well as its metabolites lysoPA and diacylglycerol (DAG) are important second messengers and are thought to regulate different cellular functions^4^. Two isoforms of PLD have been identified: PLD1 has a low basal activity and is readily activated by PKC and small GTPases of the adenosine diphosphate (ADP)-ribosylation factor (ARF) and Rho family, while PLD2 shows a high basal activity and is only marginally induced by a variety of activators. PLD plays an important role in neutrophil chemotaxis, cell migration, and modulates integrin-mediated cell adhesion^5,6^ and is associated with different diseases such as cancer, influenza, hypertension and neurodegenerative disease^7^, coronary heart disease^8–10^ and arterial thrombosis^11^. PLD1 deficiency in mice led to impaired activation of integrin αIIbβ3 and defective thrombus formation under high shear conditions. Accordingly, *Pld1*^−/−^ mice are protected against arterial thrombosis and ischemic brain infarction^11^. Moreover, PLD1 plays a pivotal role in Tumor Necrosis Factor-alpha (TNF-α) mediated inflammation and scar formation after acute myocardial infarction in mice. Deficiency of PLD1 led to defective migration of inflammatory cells into the infarct zone 24 hrs. after ischemia/reperfusion injury in mice, likely owing to reduced TNF-α expression and release. After 28 days of ischemia/reperfusion injury, scar formation was altered as well leading to increased infarct size and impaired left ventricular function in *Pld1*^−/−^ mice^9^. The impact of PLD1 in inflammatory disease was also shown by others suggesting that PLD1 plays a crucial role in diseases with high inflammatory burden.

A role for PLD2 in vascular, immunological, and neurologic disease has been shown by different groups^12^. In sepsis, PLD2 deficiency increased survival of mice and diminished organ damage during sepsis. This effect was related to decreased release of inflammatory cytokines such as TNF-α, IL-1β, IL-17 and reduced apoptosis in kidney and liver^13^. However, the function and regulation of PLD1 in sepsis has not been explored to date.

In this study we show that PLD1 is crucial for the TNF-α induced inflammatory response after LPS-induced sepsis in mice thereby regulating TNF-α expression and release, cell survival and thrombin generation and fibrin formation in septic mice.

## Materials and Methods

### Ethic statement

All animal experiments were conducted according to the Declaration of Helsinki and German law for the welfare of animals. The protocol was approved by the Heinrich Heine University Animal Care Committee and by the district government of North Rhine-Westphalia (LANUV).

### Chemicals and antibodies

Apyrase (Grade III, from potatoe). Cy™5 Annexin V (#559933) and mouse anti-human CD42a (#558819), FITC hamster anti mouse CD45 (#553252) and rat anti mouse Ly-6G (#551459) were from BD Biosciences. FITC hamster anti mouse CD40L (#ab24934) was from abcam, and FITC Mouse anti human FasL (#ab87023) was from abcam, PE rat anti mouse JON/A (integrin αIIbβ3, M023-2), FITC rat anti mouse CD62P (#M130-1), PE rat anti mouse GPIbα (##M040-2) and purified rat anti mouse GPIbα (#M042-0) was purchased from emfret analytics, cleaved caspase 3 (#9661), (phospho) MAPK family antibody sampler kit (#9926 and #9910) and (phospho) rabbit anti mouse MEK1/2 (#9122, #9121) was from Cell Signaling, rabbit anti mouse EGR-1 (#sc-110) was from Santa Cruz, rabbit anti human fibrin(ogen) (#2022-03) was from Dako, GP9 antibody was purchased from Bioorbyt (#orb 167288) and HRP-conjugated anti-mouse IgG (#NA931) was from GE Healthcare. All other reagents were of analytical grade.

### Animals

Pld mutant mice were described previously^9^. Gene-targeted mice lacking PLD1 (*Pld1*^−/−^) and the corresponding wild-type littermates (*Pld1*^+/+^) were bred from breeder pairs and genotyped by PCR. *Pld1*^*fl/fl*^ mice were kindly provided by Dr. Di Paolo (Columbia University Medical Center New York) and crossed to PF4-Cre mice, which were purchased from The Jackson Laboratory (C57BL/6-Tg [Pf4-cre] Q3Rsko/J). PF4-Cre^+^ Pld1^fl/fl^ mice or PF4-Cre^−^ Pld1^fl/fl^ littermate controls were analyzed for platelet-leukocyte interactions, neutrophil migration into the lung, platelet FasL exposure and cell apoptosis in liver and lungs. Experiments were performed with male and female mice aged 2–4 months.

### Sepsis mouse model

For plasma determinations, mice were intraperitoneally (i.p.) injected with the doses of 4 mg/kg bodyweight LPS (*E. coli* 0111: b4 product number L2630 Sigma, diluted in PBS). For survival assays, mice were i.p. injected with the dose of 10 mg/kg bodyweight LPS (*E. coli* 0111: b4 product number L2630 Sigma, diluted in PBS).

### Determination of blood cell counts

The number of platelets and leukocytes in whole blood of control and septic mice was measured by Sysmex. Where indicated number of neutrophils was determined by flow cytometry using Ly-6G antibody.

### Murine platelet preparation

Platelets were prepared as previously described^14,15^. Blood was taken from the retro-orbital plexus and centrifuged at 250 g for 5 minutes at room temperature. To obtain platelet-rich plasma (PRP), the supernatant was centrifuged at 50 × g for 6 min. PRP was washed twice at 650 × g for 5 min at room temperature and pellet was resuspended in Tyrode’s buffer [136 mM NaCl, 0.4 mM Na_2_HPO_4_, 2.7 mM KCl, 12 mM NaHCO_3_, 0.1% glucose, 0.35% bovine serum albumin (BSA), pH 7.4] supplemented with prostacyclin (0.5 µM) and apyrase (0.02 U/mL). Before use, platelets were resuspended in the same buffer and incubated at 37 °C for 30 min.

### Flow cytometry

Cell suspensions, blood components according to the respective experiment, were diluted with PBS to a concentration of 100.000 cells/µl. 50 µl thereof were incubated with 5 µl labeled antibodies for 20 minutes at RT. Staining was stopped by addition of 400 µl PBS and analyzed directly on a FACSCalibur (BD Bioscience). For AnnexinV-Cy5 staining Binding Buffer (10 mM Hepes, 140 mM NaCl, 2.5 mM CaCl_2_, pH 7.4) was used instead of PBS and only 4 µl AnnexinV-Cy5 were necessary. GPIbα was used as platelet specific marker. For analysis platelets were gated using their forward- and side-scatter profiles. Externalization of Fas ligand (FasL), CD62 and CD40L and the active form of αIIbβ3 integrin (JON/A-PE) on activated and non-activated platelets was determined by flow cytometry.

### Histology

Histological analyzes were performed with snap-frozen tissue. Liver and lungs were fixed in formaldehyde, and clarification, dehydration and inclusion in paraffin were carried out. The compounded paraffin-blocks were cut in sections with a thickness of 5 µm by a microtome (Microm HM400). Sections of hydrated and deparaffinised tissues were stained with hematoxylin and eosin (HE). After the staining procedures, images (magnification 25x, 100x, 400x) were obtained with a Carl Zeiss microscope used for this purpose and a AxioCam 105 Colour camera with the software Zen 2012 (blue edition, Carl Zeiss) was used for image capturing. Trichrome staining was performed according to Masson’s.

### Immunocytochemistry

Immunocytochemistry was performed using standard techniques. In lung and liver tissue, anti GP9 antibody (bioorbyt, #orb 167288) was used to detect platelets, neutrophils were detected with Ly6G antibody (BD, #551459). Primary antibodies were visualized with goat anti rabbit Alexa Fluor 568 and goat anti rat Alexa Fluor 568 secondary antibodies (Invitrogen). For platelet and neutrophil localization, an Axio Observer.D1 (Carl Zeiss) was used. To determine active caspase-3, anti caspase 3 antibody (Cell Signaling, #9661,) was used and visualized with the secondary antibody donkey anti rabbit Alexa Fluor 555 (Thermo Fisher, #31572) to detect cell apoptosis in lung and liver tissue. Fibrin(ogen) staining was performed using anti human fibrin(ogen) antibody (Dako, #2022-03) that was visualized with the secondary antibody goat anti rabbit Alexa fluor 488 (Invitrogen).

### Enzyme-linked Immunosorbent Assay (ELISA)

TNF-α and IL-6 levels in the supernatant of mouse embryonic fibroblasts (MEFs) and in plasma of septic mice were determined by specific ELISA (BD Pharmingen) following the manufacturer’s protocol.

### Fibrinogen levels in plasma

Determination of fibrinogen levels in citrated plasma was performed according to Clauss using the automated instrument BCS XP (Siemens).

### RT-PCR

cDNA purification and RT-qPCR analysis of TNF-α, Egr-1, Bax, Bcl-2 and Bcl-XL were performed as previously described^16^. The following primer sequences were used: for 5′-GCCCCCACTCTGACCCCTTT-3′ and rev 5′-GGGGCTGGCTCTGTGAGGAA-3′ (TNF-α), for 5′-TCCTCTCCATCACATGCCTG-3′ and rev 5′-CACTCTGACACATGCTCCAG-3′ (Egr-1), for 5′-TGAAGACAGGGGCCTTTTTG-3′ and rev 5′-AATTCGCCGGAGACACTCG-3′ (Bax), for 5′-ATGTGTGTGGAGAGCGTCAA-3′ and rev 5′-CATGCTGGGGCCATATAGTT-3′ (Bcl-2), for 5′-GACAAGGAGATGCAGGTATTGG-3′ and rev 5′-TCCCGTAGAGATCCACAAAAGT-3′ (Bcl-XL). cDNA was purified from the liver of *Pld1*^+/+^ and *Pld1*^−/−^ mice treated with or without 100 µg LPS at indicated time-points.

### Liver enzymes

Levels of the liver enzymes aspartate aminotransferase (AST), alanine aminotransferase (ALT) and lactate dehydrogenase (LDH) were determined in serum from *Pld1*^+/+^ and *Pld1*^−/−^ mice using Spotchem-biochemical analyzer EZ SP-4430 (AxionLaB/Arkray).

### Cell culture

For cultivating *Pld1*^−/−^ and *Pld1*^+/+^ MEFs, pregnant mice were sacrificed at day 13 or 14 post-coitum by cervical dislocation, embryos were separated from placenta and embryonic sac, head and red organs were dissected and cells were isolated by mincing and treatment with 0.05% trypsin EDTA. The cells were cultured in DMEM-medium (Gibco) containing 10% FCS, 1% penicillin/streptomycin, 1% nonessential amino acids and 0,2% gentamicin at 37 °C in an atmosphere of 5% CO_2_. For detachment 0.05% trypsin-EDTA (Life Technologies) was used during passaging.

### Survival (caspase 3/7) assay

For measurement of caspase 3/7 in samples of LPS stimulated MEFs the caspase Glo 3/7 assay by Promega was used following the manufacturer’s manual. 10^4^ MEFs were used per sample and samples have been stimulated with either 1 µg LPS/ml or 2 µg LPS/ml.

### Thrombin generation (calibrated automated thrombography). Thrombin generation capacity in whole blood and platelet-poor plasma

The calibrated automated thrombography was used to assess thrombin generation in citrated whole blood and platelet-poor plasma. Quantification of thrombin formation in citrated whole blood was performed according to Ninivaggi M *et al*. and Jurk K *et al*. with some modifications^17,18^. Briefly, rhodamine-based thrombin substrate P_2_Rho (final concentration 300 µM) and α-thrombin (final concentration 0.1 U/ml)) were added to 30 µl of citrated whole blood. Thrombin generation was started by adding CaCl_2_ (final concentration 17 mM) in HEPES-buffer containing 0.5% BSA. Filter paper discs (5 mm diameter and 180 µm thickness) were wetted with 5 µl of the blood mixture and covered with 40 µl mineral oil. Thrombin-mediated cleavage of the rhodamine-based substrate P2Rho was monitored for 60 minutes at 37 °C with a Fluoroskan Ascent fluorescence reader (excitation 485 nm, emission 538 nm wavelengths). Further, thrombin generation was quantified in PPP from *Pld1*^+/+^ and *Pld1*^−/−^ mice triggered by tissue factor (5 pM tissue factor plus phospholipids, PPP-reagent (Thrombinoscope/Stago)) as described^19^. Thrombin-mediated cleavage of the fluorogenic substrate Z-Gly-Gly-Arg-AMC was monitored for 60 minutes at 37 °C using a Fluoroskan Ascent fluorescence reader (excitation 390 nm, emission 460 nm wavelengths, Thermo Labsystems, Franklin, MA). The ThrombinoscopeTM, Synapse BV software program was used for calculation of thrombin generation parameters.

### Cell lysis and immunoblotting of MAPKs, MEK1/2 and EGR-1

Western blot analysis were performed to compare the protein expression of the MAPK (mitogen-activated protein kinases) pathway after LPS (*e. coli* 0111: b4 product number L2630 Sigma, diluted in PBS) stimulation of Pld1^−/−^ and Pld1^+/+^ MEFs. Therefor 5*10^4^ MEFs per well were seeded on 48 well plates, incubated for 3 h and stimulated with either LPS (1 µg/ml) or PBS for 30 minutes. Cells were lysed with the following buffer: 15 mM Tris-HCl; 155 mM NaCl; 1 mM EDTA; 0,005% NaN_3_; 1% IGPAL; 1 mM Na_3_VO_4_; Proteaseinhibitor.

As primary antibodies (phospho-) ERK1/2, SAPK/JNK and p38 MAPK ((phospho) MAPK family antibody sampler kit (#9926 and #9910), MEK1/2 (CellSignaling, #9122), phospho-MEK1/2 (CellSignaling, #9121) ERK1/2 (Cell Signaling #4695), phospho-ERK1/2 (Cell Signaling #4370) and Egr-1(Santa Cruz) were used. The cell lysates were prepared with Laemmli buffer, denaturated for 5 minutes at 95 °C and separated on SDS- polyacrylamide gel. The proteins were transferred onto nitrocellulose blotting membranes, which were blocked with TBST containing 5% dried milk. The antibody incubation was performed following the manufacturer’s manual and incubated with peroxidase-conjugated anti-rabbit IgGs (GE Healthcare, Code: NA9340, 1:2500). For visualizing protein bands Immobilon Western Chemiluminescent HRP Substrate solution (BioRad) was used.

### Statistical analysis

Data are demonstrated as mean ± standard error of the mean (s.e.m.). Statistical significance was analyzed by Student’s paired t-test and by Log-rank Mantel-Cox test. P values < 0.05 were considered to be statistically significant.

## Results

### PLD1 deficiency decreases mortality in LPS-induced sepsis by modulating TNF-α expression and release

**Figure 1:**
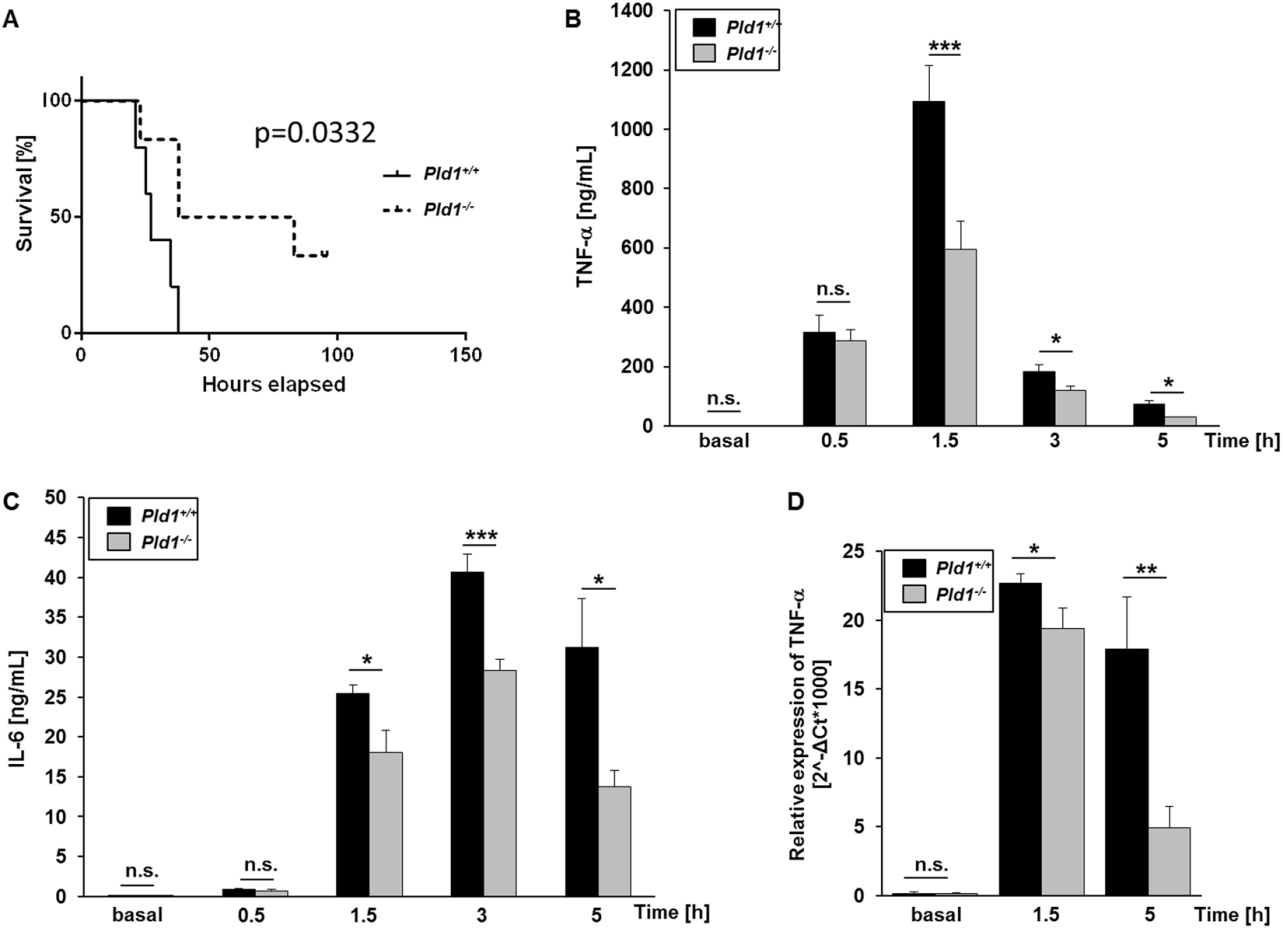
PLD1 deficiency decreases mortality in LPS-induced sepsis by modulating TNF-α expression and release. (**A**) *Pld1*^+/+^ and *Pld1*^−/−^ mice were injected with 10 mg/kg bodyweight LPS and survival was monitored for 100 hrs. N = 10 per group. P-value = 0.0332, Log-rank Mantel-Cox test. (**B,C**) *Pld1*^+/+^ and *Pld1*^−/−^ mice were injected with 4 mg/kg bodyweight LPS and serum TNF-α (**B**) and IL-6 (**C**) were measured at indicated time points after LPS injection and under basal conditions using ELISA. (**D**) TNF-α expression in liver tissue was analyzed by qRT-PCR at indicated time points following LPS injection. N = 5 (**B**–**D**). Bar graphs depict mean values ± s.e.m. *P < 0.05, **P < 0.01, ***P < 0.001.

TNF-α is a major factor in endotoxin^20,21^ and super antigen toxicity^22^. PLD1 has been shown to be involved in TNF-α expression and release upon myocardial ischemia and reperfusion injury. To investigate the role of PLD1 in septic shock, *Pld1*^−/−^ mice were challenged with bacterial LPS. Whereas most of the wildtype (*Pld1*^+/+^) mice died within 48 hrs., numerous *Pld1*^−/−^ mice were resistant to a dose of 250 µg LPS per mouse showing improved survival after LPS challenge (Fig. 1A, p = 0.0332). To examine if PLD1 regulates TNF-α expression and release after septic shock as observed after acute myocardial infarction^9^, we injected *Pld1*^+/+^ and *Pld1*^−/−^ mice with LPS (4 mg/kg bodyweight) and determined TNF-α plasma levels. The mutant mice showed significantly less serum TNF-α than *Pld1*^+/+^ mice 1.5, 3 and 5 hrs. after LPS challenge (Fig. 1B). Concomitant with reduced TNF-α levels, serum IL-6 was significantly reduced after 1.5, 3 and 5 hrs. after LPS injection as well (Fig. 1C). Furthermore, TNF-α expression in liver after LPS challenge was significantly reduced in *Pld1*^−/−^ mice as determined by RT-PCR 1.5 and 5 hrs. after LPS injection (Fig. 1D).

### PLD1 modulates TNF-α expression and release via phosphorylation of MEK1 and ERK1/2

**Figure 2:**
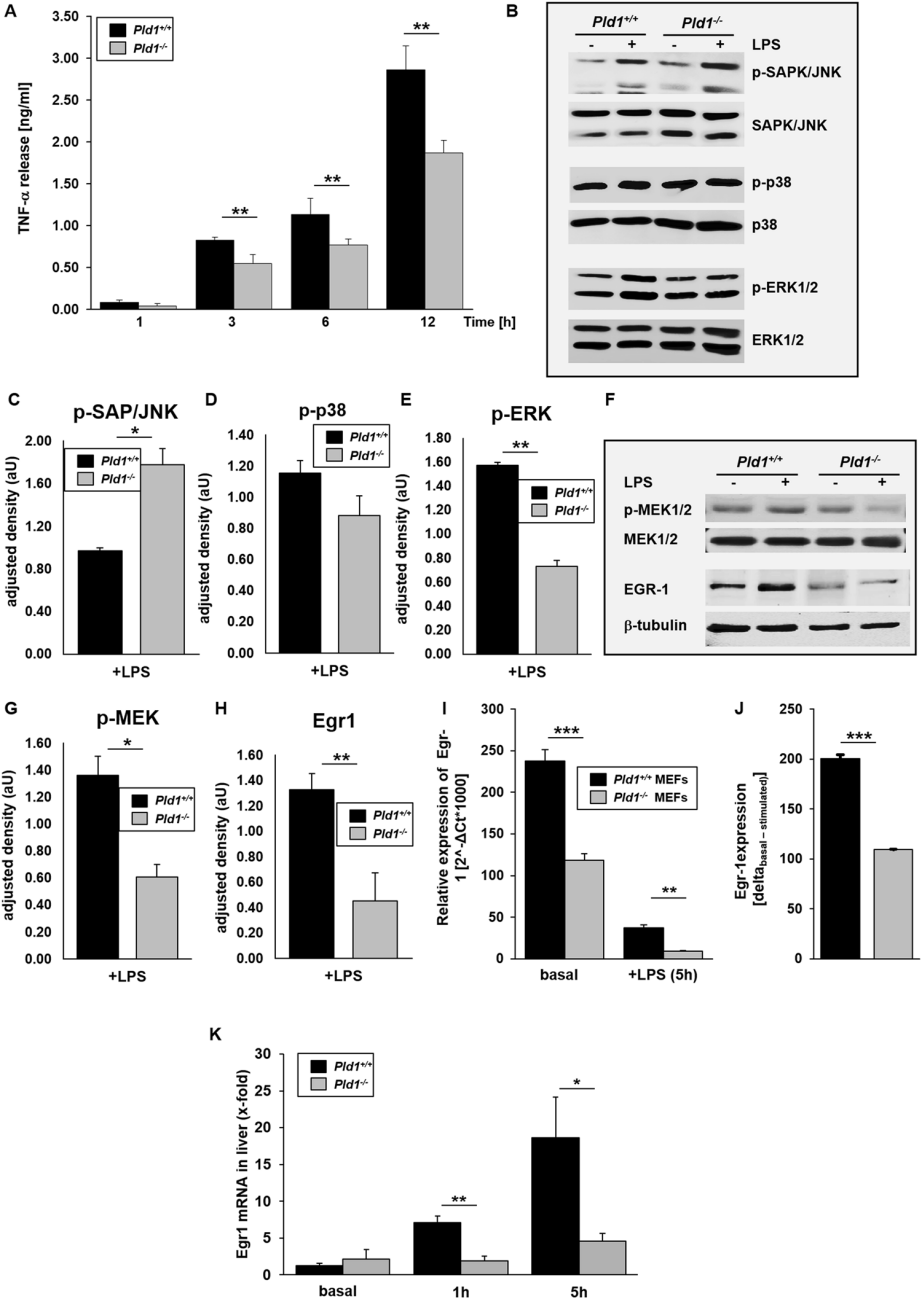
PLD1 modulates TNF-α expression and release via phosphorylation of MEK1/2 and ERK1/2. MEFs from *Pld1*^+/+^ and *Pld1*^−/−^ mice were stimulated with 1 mg/ml LPS for indicated time points. (**A)** TNF-α release into the supernatant of MEFs after LPS stimulation was measured by ELISA. (**B**–**E**) Phosphorylation of SAPK/JNK, p38 and ERK1/2 was detected by Western blot after stimulation of MEFs with LPS for 30 min. and quantified via adjusted density using ImageJ. Same samples for quantification of total protein expression were used but applied to different gels/membranes. Cropped blots are shown. (**F**–**H**) Phosphorylation of MEK1/2 and protein abundance of EGR-1 was detected after LPS stimulation of MEFs for 30 min. using Western blot analysis and quantified via adjusted density using ImageJ. B-tubulin serves as loading control for the detection of EGR-1. Cropped blots are shown. (**I**) Egr-1 expression in MEFs was analyzed by qRT-PCR 5 hrs. after LPS stimulation. (**J**) ΔEgr-1 expression (basal-stimulated) of *Pld1*^+/+^ and *Pld1*^−/−^ MEFs. N = 5. (**K**) Egr-1 expression in the liver of PLD1 deficient and control mice was determined by qRT-PCR. N = 9. Bar graphs depict mean values ± s.e.m. *P < 0.05, **P < 0.01 ***P < 0.001.

To investigate how PLD1 controls TNF-α expression, we established a primary cell culture by the generation of mouse embryonic fibroblasts (MEFs) from *Pld1*^+/+^ and *Pld1*^−/−^ mice and determined TNF-α release after LPS stimulation of cells (Fig. 2A). MEFs from *Pld1*^−/−^ mice exhibited decreased LPS-induced TNF-α levels in the supernatant after all time points analyzed (1, 3, 6 and 12 hrs. after LPS addition). The signaling cascade of LPS binding to toll-like receptor (TLR)4 is well known. LPS binding to TLR4 triggers the activation of MAP kinase kinase kinase (MAP3K) including MEK1/2, MKK4/7, MKK3/6 leading to the phosphorylation of ERK1/2, SAPK/JNK and p38^23^. To determine the potential mechanism by which PLD1 might control TNF-α expression, MEFs were stimulated with LPS for 30 min. and the phosphorylation of SAPK/JNK, p38 and ERK1/2 was analyzed in MEF lysates. As shown in Fig. 2B–D, LPS stimulation of cells led to increased phosphorylation of SAPK/JNK and p38 in both, *Pld1*^+/+^ and *Pld1*^−/−^ MEFs whereas the phosphorylation of ERK1/2 was increased only in MEFs from *Pld1*^+/+^ mice but not in *Pld1*^−/−^ MEFs (Fig. 2B,E) suggesting that PLD1 regulates TNF-α expression via the LPS-TLR4-MAP3K-ERK1/2 pathway. However, when we analyzed the phosphorylation of the upstream kinase MEK1/2 we already found reduced phosphorylation of this kinase in *Pld1*^−/−^ MEFs, suggesting that PLD1 might regulate the MAP3K MEK1/2 in the LPS-TLR4 pathway (Fig. 2F,G). Protein expression of Egr-1 is induced by the phosphorylation of MAP3Ks leading to the activation of AP-1 via binding to transcription factor (TF) promoter to induce TNF-α expression^24,25^. According to reduced MEK1/2 phosphorylation in *Pld1*^−/−^ MEFs, EGR-1 protein was only marginally detectable while a strong signal of EGR-1 was detected in MEFs from *Pld1*^+/+^ mice following LPS challenge using Western blot (Fig. 2F,H). Egr-1 protein expression was confirmed by RT-PCR showing enhanced Egr-1 expression in *Pld1*^+/+^ MEFs but low expression in *Pld1*^−/−^ MEFs in absolute levels and when we determined the difference (delta) of basal to LPS stimulated levels of Egr-1 (Fig. 2I,J). Egr1 mRNA (x-fold) was also determined in the liver of *Pld1*^+/+^ and *Pld1*^−/−^ mice under basal conditions and in LPS treated mice showing significant differences 1 h and 5 h after LPS injection (Fig. 2K).

### Lack of PLD1 prevents LPS-induced liver and lung damage in mice

**Figure 3:**
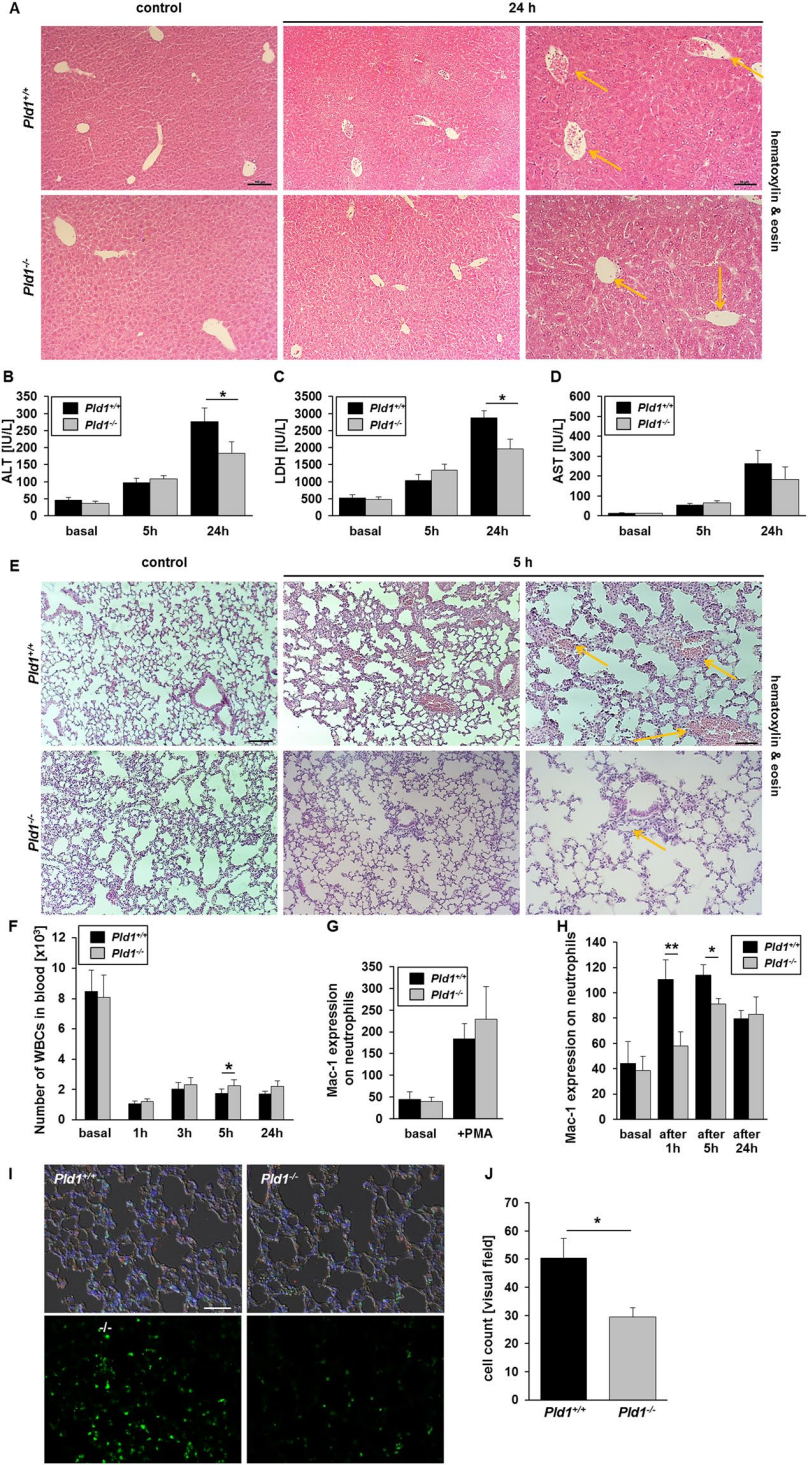
Loss of PLD1 prevents LPS-induced liver and lung damage in mice. (**A**) *Pld1*^+/+^ and *Pld1*^−/−^ mice were i.p. injected with 4 mg/kg bodyweight LPS. Livers were snap-frozen 24 h after LPS injection and sections were stained with hematoxylin & eosin (H&E). Yellow arrows indicate (occluded) vessels. N = 5, scale bar = 100 µm. (**B**–**D**) Liver enzymes such as ALT, AST and LDH were measured 5 and 24 hrs. after LPS injection in plasma of *Pld1*^+/+^ and *Pld1*^−/−^ mice and compared to basal levels. (**E**) 5 hrs. after LPS treatment of mice, lungs were snap-frozen and sections were stained with H&E. Yellow arrows indicate (occluded) vessels. N = 4, scale bar = 100 µm. (**F**) Number of white blood cells (WBCs) in blood of *Pld1*^+/+^ and *Pld1*^−/−^ mice at indicated time points were measured by Sysmex cell counter. 5 h after LPS injection, significantly increased numbers of WBCs were measured in PLD1 deficient mice (p = 0.025). (**G**,**H**) Mac-1 expression at the plasma membrane of neutrophils was measured by flow cytometry. (**G**) No differences were determined using neutrophils form healthy *Pld1*^+/+^ and *Pld1*^−/−^ mice. (**H**) Mac-1 expression was altered in septic mice after indicated time points. **(I,J**) Neutrophil recruitment in lungs of *Pld1*^+/+^ and *Pld1*^−/−^ mice 5 h after LPS injection. (**I**) Neutrophils were stained with Ly6G (green). Nuclei were stained with 4′,6′-diamidino-2-phenylindole (DAPI). Merge included staining of platelets with GP9 (red) and is shown in Differential Interference Contrast (DIC) mode. (**J**) Number of neutrophils migrated into lungs was quantified per visual field. N = 4, scale bar = 50 µm.

We next examined liver histology 5 and 24 hrs. after injection of LPS. As shown in Fig. 3A, LPS caused marked, time-dependent liver damage with disrupted liver architecture 24 hrs. after LPS injection in *Pld1*^+/+^ but not in *Pld1*^−/−^ mice (Fig. 3A). Marker of liver damage such as alanine transaminase (ALT) and lactate dehydrogenase (LDH) were also significantly elevated in *Pld1*^+/+^ mice 24 hrs. after LPS injection compared with *Pld1*^−/−^ mice whereas no differences were detected in the elevation of aspartate transaminase (AST) (Fig. 3B–D).

Histological analysis of lungs by hematoxylin-eosin (HE) staining showed less inflammatory cell infiltration, alveolar congestion and thrombotic lesions in *Pld1*^−/−^ compared to *Pld1*^+/+^ mice (Fig. 3E). We next examined pulmonary infiltration of neutrophils in the lung by Ly6G staining (Fig. 3I). In *Pld1*^+/+^ mice we observed large-scale infiltration of neutrophils into the lung whereas less neutrophil infiltration was found in *Pld1*^−/−^ mice. Accordingly, the number of leukocytes in the blood stream was significantly enhanced in *Pld1*^−/−^ mice 5 hrs. after LPS challenge (Fig. 3F). Moreover, the expression of Mac-1 on neutrophils was significantly reduced in *Pld1*^−/−^ mice 1 and 5 hrs. after LPS injection whereas no differences were detected when neutrophils were isolated from healthy mice and analyzed for Mac-1 expression after PMA stimulation as control (Fig. 3G,H). Mac-1 expression is important for neutrophil extravasation, thus neutrophil recruitment into lungs of *Pld1*^+/+^ and *Pld1*^−/−^ mice 5 h after LPS injection were examined. As shown in Fig. 3I,J, the number of neutrophils that migrated into the lung is significantly reduced in PLD1 deficient mice compared to controls (Fig. 3I,J). The analysis of *Pld1*^*fl/fl*^-PF4 cre mice with specific deletion of PLD1 only in platelets revealed that differenced in WBC count, Mac1 expression and invasion of neutrophils into the lung of septic animals is not depending on platelet-leukocyte interaction (Suppl. Fig. 1A–F).

### PLD1 deficiency leads to reduced cell apoptosis

**Figure 4:**
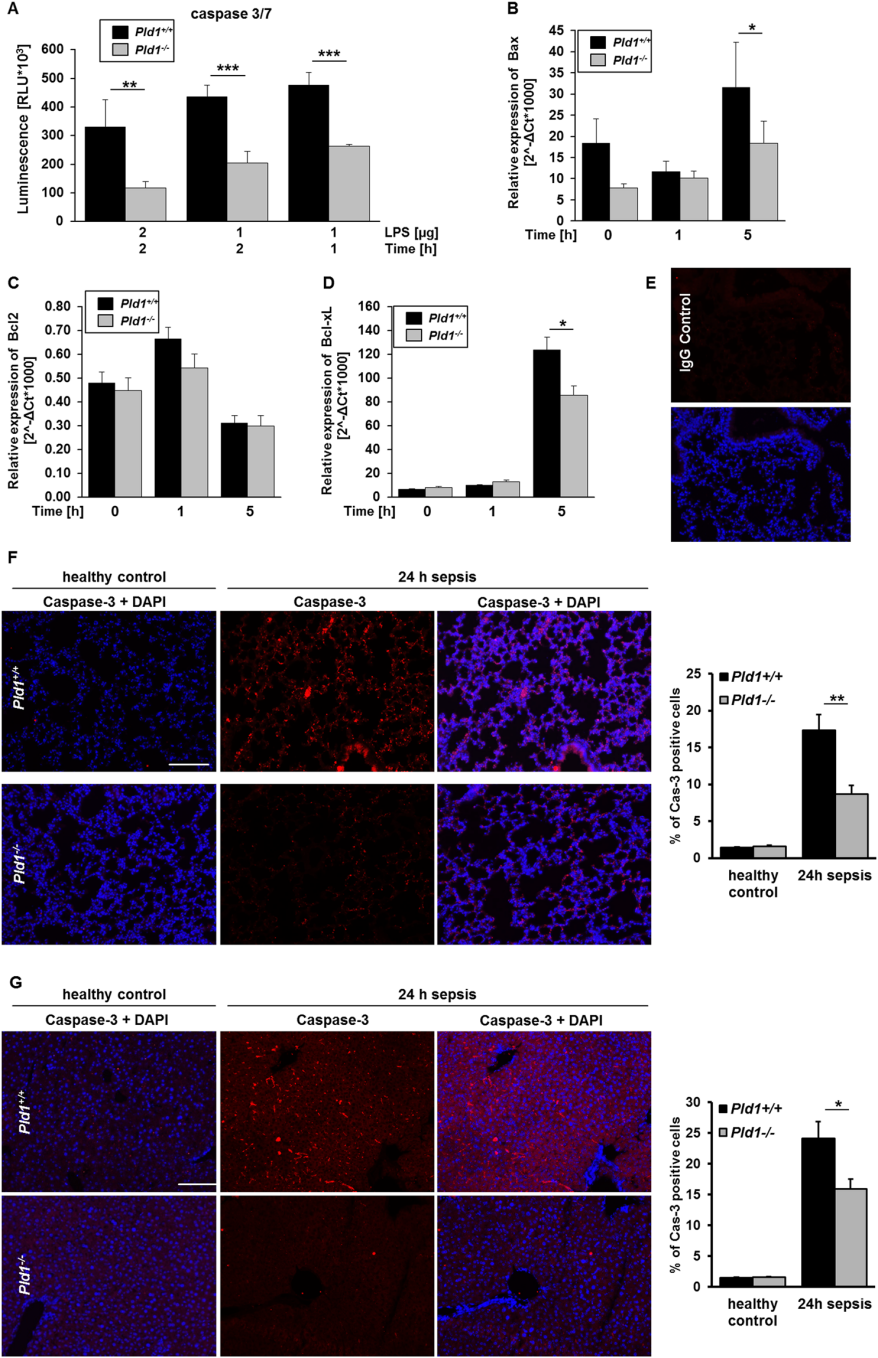
PLD1 deficiency in mice leads to reduced cell apoptosis. (**A**) Survival (caspase 3/7) of MEFs from *Pld1*^+/+^ and *Pld1*^−/−^ mice was determined at indicated time points after stimulation with 1 and 2 µg LPS, respectively. N = 4. (**B**–**D**) Expression of pro- and anti-apoptotic markers in liver was determined by qRT-PCR. RNA levels of Bax (**B**) (p = 0.038), Bcl2 (**C**) and Bcl-xL (**D**) (p = 0.021) after indicated time points is shown. N = 5. (**E–G**) Lung (**E,F**) and liver (**G**) sections from healthy (left) and septic (right) *Pld1*^+/+^ and *Pld1*^−/−^ mice were stained with active caspase 3 antibody (red) and visualized by immunofluorescence microscopy (left panel). Nuclei were stained with DAPI. (**F,G**) The number of caspase-3 positive cells was determined (right panel). N = 5, scale bar 100 µm. Data are expressed as arithmetic means ± s.e.m. *P < 0.05, **P < 0.01, ***P < 0.001.

We next investigated the potential of PLD1 to modulate cell survival and apoptosis after LPS induced sepsis. First, the survival of MEFs after LPS stimulation was analyzed. Determination of active caspase 3/7 in LPS-stimulated MEFs revealed significantly reduced activation of caspase 3 and 7 at different time points and LPS concentrations (Fig. 4A). According to reduced levels of  active caspases in PLD1 deficient MEFs, the expression of pro- and anti-apoptotic proteins in the liver of *Pld1*^−/−^ mice was analyzed and compared to controls. In detail, the expression of the pro-apoptotic protein Bax and the anti-apoptotic protein Bcl-xL were significantly reduced in *Pld1*^−/−^ livers 5 hrs. after LPS injection compared to controls (Fig. 4B–D). In line with these results, we observed less active caspase 3 positive cells in lungs and livers 24 hrs. after LPS-induced sepsis in *Pld1*^−/−^ mice compared to *Pld1*^+/+^ controls (Fig. 4E–G).

### PLD1 deficient platelets contribute to preserved outcome of *Pld1*^−/−^ mice after LPS-induced sepsis

**Figure 5:**
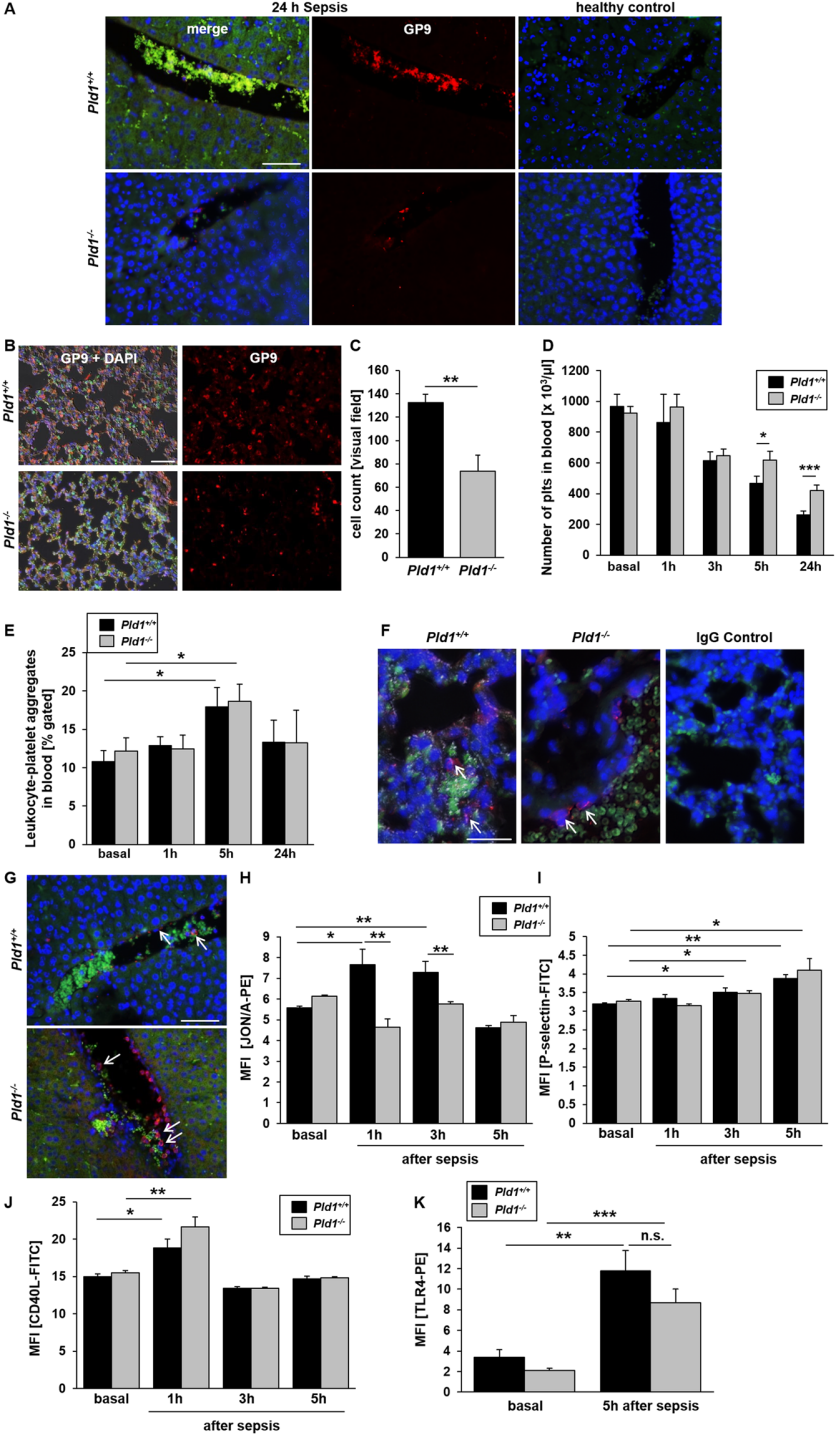
PLD1 deficient platelets contribute to preserved outcome of *Pld1*^−/−^ mice after LPS-induced sepsis. *Pld1*^+/+^ and *Pld1*^−/−^ mice were i.p. injected with 4 mg/kg bodyweight LPS. (**A,B**) Liver (**A**) and lung (**B**) sections from healthy and septic *Pld1*^+/+^ and *Pld1*^−/−^ mice were stained with GP9 antibody to visualize platelets (red) in liver (24 hrs. after LPS injection) and lung (5 hrs. after LPS injection) tissue. (**A**) In liver, red blood cells show auto fluorescence (green). Nuclei were stained with DAPI. (**B,C**) In lungs, neutrophils were stained with Ly6G (green) and nuclei with DAPI. Platelets were quantified per visual field. Merge in Differential Interference Contrast (DIC) mode. N = 5, scale bar = 100 µm (liver) and 50 µm (lung). (**D**) Number of platelets in blood from healthy and septic mice after indicated time points as measured by the cell counter Sysmex. N = 15. (**E**) Platelet-leukocyte conjugates basal and after LPS injection were measured by flow cytometry using leukocyte specific antibody CD45 and platelet marker GPIbα. N = 5. (**F,G**) Leukocyte-platelet aggregates were stained and visualized by immunofluorescence microscopy in lung (**F**) and liver (**G**) of septic mice 24 hrs. after LPS injection. GP9 antibody stains platelets (red), nuclei were stained with 4′,6′-diamidino-2-phenylindole (DAPI). N = 5, scale bar = 50 µm (lung) and 100 µm (liver). (**H,I**) Platelet activation by determination of active integrin αIIbβ3 (JON/A) and P-selectin exposure as marker for degranulation was measured by flow cytometry using whole blood from septic mice. (**J**) CD40L exposure of isolated platelets from septic *Pld1*^+/+^ and *Pld1*^−/−^ mice was measured by flow cytometry. (**K**) TLR4 expression at the platelet surface was measured in heatlhy and septic mice 5 h post LPS injection. N = 7. Data are mean ± s.e.m. *P < 0.05, **P < 0.01, ***P < 0.001, n.s. not significant.

As important effectors of both, thrombosis and inflammation, platelets are involved in sepsis pathogenesis and contribute to sepsis complications whereas platelet activation in sepsis correlates with organ dysfunction^26,27^. In healthy mice, PLD1 deficiency protects against arterial thrombosis after vessel injury because of a defect in integrin αIIbβ3 (fibrinogen receptor) activation leading to reduced thrombus formation under high shear conditions^11^. Moreover, PLD1 deficient platelets showed less endothelial and leukocyte activation upon inflammation^28^. Therefore, we wanted to know if PLD1 deficient platelets contribute to improved survival of mice after LPS-induced sepsis. Examination of liver (Fig. 5A) and lung histology (Fig. 5B,C) revealed less thrombi in liver tissue and less infiltration of platelets into lungs of *Pld1*^−/−^ mice compared to controls. Accordingly, the number of platelets in the blood stream was significantly enhanced 5 and 24 hrs. after LPS injection compared to corresponding LPS treated *Pld*^+/+^ mice (Fig. 5D). The number of leukocyte-platelet aggregates in the blood stream as determined by flow cytometry (Fig. 5E) as well as in lungs (Fig. 5F) and livers (Fig. 5G) as shown by immunofluorescence staining were not different between PLD1 deficient and control mice (Fig. 5E–G). This result was confirmed by the analysis of leukocyte-platelet aggregates in *Pld1*^*fl/fl*^-PF4 cre mice (Suppl. Fig. 1C–D). However, using whole blood of septic mice we detected reduced integrin αIIbβ3 activation of platelets as measured by flow cytometry using the antibody JON/A that detects only active integrin (Fig. 5H). This data indicates that PLD1 deficiency induces a platelet integrin defect not only in healthy^11^ but also in septic mice. P-selectin exposure of platelets 3 and 5 hrs. after LPS injection was increased in both septic *Pld1*^+/+^ and *Pld1*^−/−^ mice using whole blood of LPS treated mice (Fig. 5I).

Platelet adhesion via integrin αIIbβ3 leads to up-regulation of CD40 ligand (CD40L, CD154) at the plasma membrane of platelets and induces endothelial cells to release chemokines and to express adhesion molecules via binding to CD40 on endothelial cells^29^. The activation of the glycoproteins (GP) Ib and VI on platelets leads to reduced CD40L exposure of *Pld1*^−/−^ platelets^28^. Therefore we next determined CD40L on platelets from septic mice. Although we measured a significant increase in CD40L on the platelet membrane there were no differences between *Pld1*^+/+^ and *Pld1*^−/−^ mice detectable (Fig. 5J). Platelets expose Fas ligand (FasL) at the plasma membrane after platelet stimulation and induce cell apoptosis in target cells^30^. To investigate if platelets contribute to reduced cell apoptosis as observed in lung and liver tissue of septic mice, we determined FasL at the platelet membrane of septic (3 h post LPS injection) and of healthy mice upon stimulation with classical agonists such as collagen-related peptide (CRP) that activates the major collagen receptor GPVI, and adenosine diphosphate (ADP) (Suppl. Fig. 2A). FasL exposure was reduced in platelets from *Pld1*^*fl/fl*^-PF4-Cre+ mice compared to controls. However, reduced cell apoptosis observed in PLD1 deficient mice was not due to platelet FasL externalization (Suppl. Fig. 2A) because no differences in active caspase 3 positive cells in lungs and livers 24 hrs. after LPS-induced sepsis was observed in *Pld1*^*fl/fl*^-PF4-Cre mice (Suppl. Fig. 2B–E). However, control experiments showed unaltered TLR4 expression at the platelet surface of PLD1 deficient mice.

### PLD1 is important for thrombin generation and fibrin deposition

Platelets catalyze the development of DIC with massive thrombin formation because platelets provide a pro-coagulant surface by exposing phosphatidyl serine at the plasma membrane^26^. Thrombin is then able to convert fibrinogen into fibrin and to activate platelets^31^.

**Figure 6:**
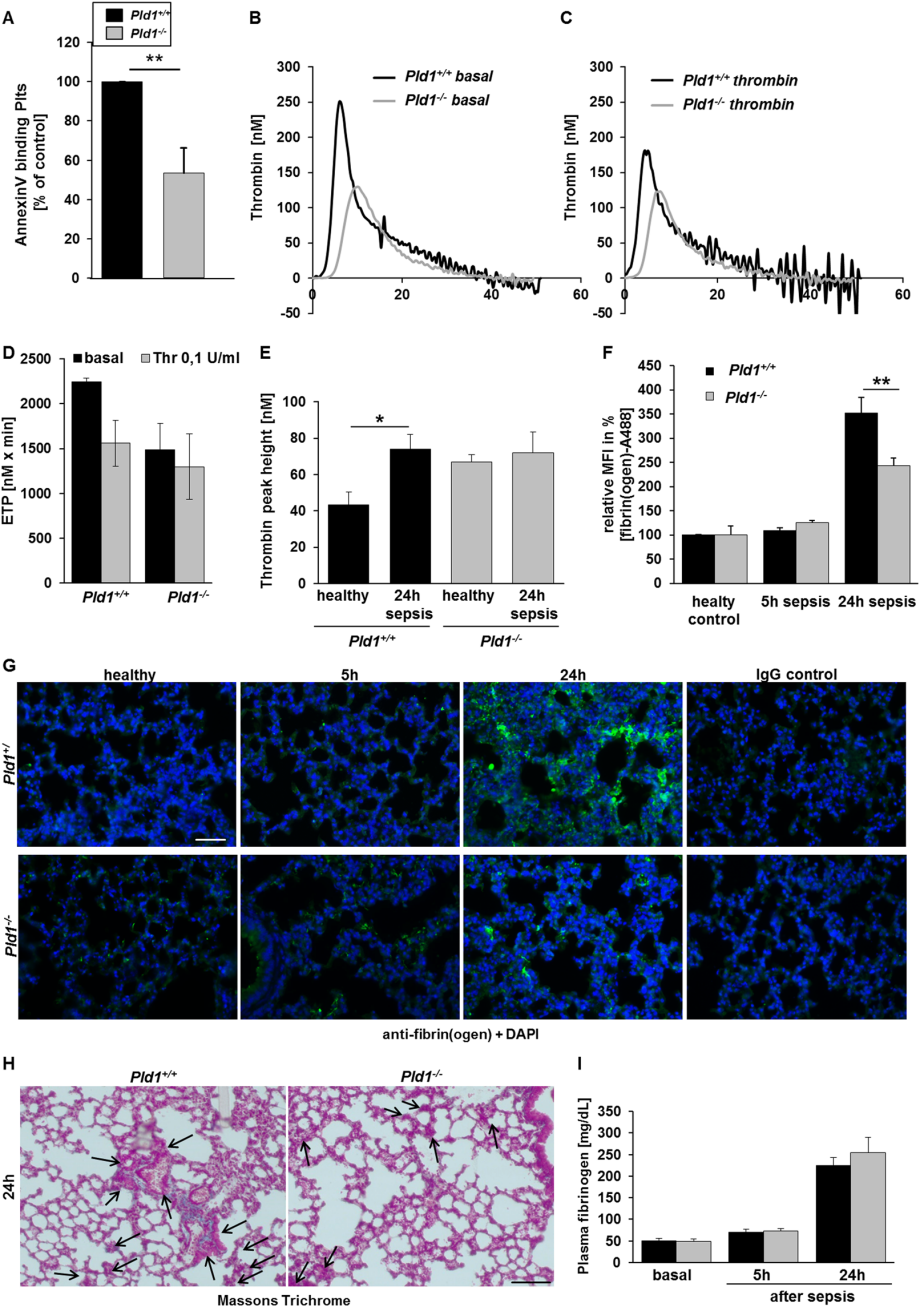
PLD1 is important for thrombin generation and fibrin deposition. (**A**) Thrombus formation on collagen under flow was performed and AnnexinV binding of platelets was measured after detaching the cells from the collagen matrix using flow cytometry. N = 6. (**B**–**D**) Basal and thrombin-induced thrombin generation in citrated whole blood of *Pld1*^+/+^ and *Pld1*^−/−^ mice was measured with the fluorogenic-calibrated automated thrombogram assay. (**B,C**) Representative curves of thrombin levels and (**D**) endogenous thrombin potential (ETP, nM x min) are shown. N = 5. (**E**) Peak height (nM thrombin) in platelet-poor-plasma (PPP) supplemented with 5 pM tissue factor and phospholipids was determined to check if plasma factors in *Pld1*^+/+^ and *Pld1*^−/−^ mice are altered per se. N = 5. (**F,G**) Lung sections from healthy (left) and septic (right) *Pld1*^+/+^ and *Pld1*^−/−^ mice were stained with fibrin(ogen) antibody (green), visualized by immunofluorescence microscopy and quantified. (**H**) Trichrome staining according to Masson’s was performed in lung sections of *Pld1*^+/+^ and *Pld1*^−/−^ mice 24 hrs. after LPS injection. N = 5, scale bar = 50 µm (**G,H**). (**I**) Fibrinogen plasma levels in *Pld1*^−/−^ and *Pld1*^+/+^ mice were measured according to Clauss. N = 5. Data are mean ± s.e.m. *P < 0.05, **P < 0.01, ***P < 0.001.

Recent data from our group indicated a contribution of PLD1 to platelet procoagulant activity^11^. In flow chamber experiments, adherent and aggregated platelets were stained with OG488–annexin showing significantly reduced annexin A5 positive platelets in thrombi formed under flow conditions. Here, we repeated flow chamber experiments and measured AnnexinV binding of platelets after detaching the cells from the collagen matrix using flow cytometry. As shown in Fig. 6A, significantly reduced PS exposure of platelets was measured when we performed experiments with whole blood from *Pld1*^−/−^ mice (Fig. 6A). To provide evidence that reduced PS exposure of PLD1 deficient platelets is responsible for reduced thrombin generation, we determined thrombin generation in citrated whole blood from healthy *Pld1*^−/−^ and *Pld1*^+/+^ mice to analyze the ability of PLD1 deficient platelets to contribute to thrombin formation in septic mice using the fluorogenic-calibrated automated thrombogram (CAT) assay. As shown in Fig. 6B–D, thrombin levels under basal conditions were reduced as confirmed by the endogenous thrombin potential (ETP) using citrated whole blood of *Pld1*^−/−^ mice compared to controls (Fig. 6B–D) and to a lesser extent after stimulation of platelets with thrombin (Fig. 6C). When we measured the capacity of coagulation factors to induce thrombin formation using platelet-poor-plasma (PPP), tissue factor and phospholipids, an increase in thrombin (peak nM) was detected in *Pld1*^+/+^ mice 24 hrs. after LPS injection but no significant differences were observed between healthy and septic *Pld1*^−/−^ mice. Interestingly, healthy *Pld1*^−/−^ mice displayed an enhanced capacity for thrombin formation (Fig. 6E) because we detected enhanced thrombin in platelet-poor-plasma (PPP) already under basal conditions.

According to these results, less fibrin(ogen) was detected in lung sections of *Pld1*^−/−^ compared to *Pld1*^+/+^ mice (Fig. 6F,G). To confirm that reduced fibrin(ogen) accumulation in lungs is due to platelet-mediated PS exposure and subsequent thrombin generation, we analyzed fibrin(ogen) in lungs of *Pld1*^*fl/fl*^-PF4-Cre+ and control mice. As expected we again found reduced fibrin(ogen) formation also in mice where only platelets are deficient of PLD1 (Suppl. Fig. 3). Masson’s Trichrome staining confirmed the results obtained with immunohistochemistry showing less fibrin accumulation in lungs of *Pld1*^−/−^ mice (Fig. 6H). However, fibrinogen plasma levels were comparable in *Pld1*^−/−^ and *Pld1*^+/+^ mice as measured according to Clauss (Fig. 6I).

## Discussion

In this study we have shown that PLD1 deficiency in mice results in a significant survival advantage after LPS-induced sepsis. Our data provide evidence for reduced TNF-α expression and release, accompanied by reduced migration of leukocytes and platelets into lung and liver, and reduced organ damage by decreased intra-organ thrombosis and cell apoptosis in liver and lung that all account for decreased mortality of PLD1 deficient mice. Moreover, integrin αIIbβ3 defects of PLD1 deficient platelets and reduced PS exposure followed by decreased thrombin generation might contribute to preserved outcome of septic PLD1 deficient mice.

TNF-α is a key mediator of septic shock and plays an important role as major factor in endotoxin^20,21^ and super antigen toxicity^22^. Mice are protected against lethality when TNF activity was antagonized with TNF antibodies or soluble human TNFRs^22,32–34^. Mice deficient for the TNF receptor TNFRp55 have been shown to be resistant to endotoxin shock because of largely abolished TNF-α signaling^35^. Regulators of TNF-α such as the rhomboid family member iRhom2 that interact with TACE to regulate TNF-α shedding showed reduced TNF-α plasma levels and enhanced survival after a lethal LPS dose^36^. This suggests that direct inhibition of TNF-α as well as altered regulation of TNF-α signaling leads to a survival benefit of mice after LPS-induced septic shock. Our data revealed that PLD1 is a key regulator of TNF-α expression upon sepsis because PLD1 plays a major role in the MEK-ERK1/2 mitogen-activated protein kinase (MAPK) pathway upon LPS induction of TNF-α gene expression. We found PLD1 to be responsible for the phosphorylation of MEK1/2 and ERK1/2 after LPS-induced TLR4 activation. LPS induction of the TNF-α gene in monocytes was already shown by Guha and colleagues who propose that activation of the MEK/ERK1/2 pathway leads to the generation of EGR-1 that binds to the TF promoter- and in association with AP-1 and c-Rel/p65- induces TF gene expression^24^. In line with these results we detected reduced amount of EGR-1 protein by Western blot and reduced Egr-1 expression after LPS stimulation of PLD1 deficient MEFs suggesting a crucial role for PLD1 in the LPS-TLR4-MEK-ERK1/2-EGR-1 axis responsible for TF expression in septic shock. A role for PLD1 in LPS-induced TNF-α expression *in vitro* was already shown by Oh and colleagues in 2014^37^. They found PLD1 to be important for LPS-TLR4 induced regulation of TNF-α expression *in vitro*. However, they used Raw 264.7 cells and proposed PLD1 mediated regulation of S6K1/JNK by knock-down strategies. In contrast we here did not observe any differences in the phosphorylation of SAPK/JNK or p38 after LPS induced activation of MEFs using PLD1 deficient cells suggesting that there might be differences according to the experimental setup or the method how to block PLD1 activity.

Different groups in the past reported about a critical role of PLD1 in TNF-α triggered inflammatory diseases such as peritonitis^38^, myocardial infarction and reperfusion injury^9^ and arthritis^39^. However, in a recent study the impact of PLD2 upon sepsis was examined^13^. PLD2 deficiency increased survival of mice and decreased vital organ damage with decreased production of inflammatory cytokines such as TNF, IL-1 and IL-17. However, the authors did not investigate TNF-α expression levels in septic mice. Using antisense oligonucleotides, Sethu and colleagues already showed in 2008 that coupling of TNF-α to the activation of ERK1/2 phosphorylation is inhibited by antisense to PLD1 but not to PLD2^40^ suggesting a specific regulation of TNF-α expression by PLD1 and not PLD2.

The PLD superfamily is discussed to be a therapeutic target for years because PLDs play a significant role in neurodegenerative disease such as Alzheimer’s disease^41,42^, hypertension^43–45^ and cancer^46^. Furthermore, PLD1 deficient mice are protected against arterial thrombosis because PLD1 is important for integrin αIIbβ3-mediated platelet activation and thrombus formation under high shear conditions^11^. A potential advantage of PLD1 inhibition is that this approach does not lead to bleeding complications in mice^47^ suggesting that PLD1 inhibition might be safer than other anti-thrombotic therapies as currently used in clinical practice.

A recent study revealed that PLD1 is also a major regulator of platelet-mediated inflammation^28^. PLD1 does not only regulate GPIb-mediated integrin activation following aggregate formation under high shear^11^ but also induces regulation of GPIb in platelet-mediated inflammation^28^ by modulating PLCγ2 phosphorylation and integrin activation via Src kinases. This suggests that PLD1 deficiency might protect against arterial thrombosis and platelet-mediated responses upon inflammation. Accordingly, increased platelet and leukocyte numbers in blood were detected whereas less neutrophils and platelets were found in lungs of septic mice. Moreover, the defective integrin αIIbβ3 activation of PLD1 deficient platelets described in the past^11,28^ were confirmed also in septic animals.

Activated αIIbβ3 integrin has been implicated in the coagulant activity of platelets^48,49^. To determine a possible role for PLD1 in phosphatidylserine exposure necessary to provide a pro-coagulant surface important for thrombin generation, we induced thrombus formation on collagen under flow and measured AnnexinV binding of platelets. As expected, significantly reduced PS exposure of platelets was measured when experiments were performed with whole blood from *Pld1*^−/−^ mice (Fig. 6E). Accordingly, the formation of thrombin was reduced because the ETP under basal condition and after stimulation of platelets with thrombin was significantly reduced in *Pld1*^−/−^ PRP compared to controls (Fig. 6A–C). Thus, thrombotic complications might be reduced in septic PLD1 deficient mice because acute DIC including massive thrombin formation and microvascular thrombosis responsible for multiple organ dysfunctions might be decreased in septic PLD1 deficient mice. Reduced intravascular aggregate formation in liver and lungs of PLD1 deficient mice was observed accordingly (Fig. 3A,E).

In the present study we observed reduced apoptosis in lungs and liver of septic PLD1 deficient mice. In line with the *in vivo* data we detected reduced active caspase 3/7 in LPS stimulated MEFs and altered expression of pro- and anti-apoptotic genes in liver of septic mice (Fig. 4). Reduced apoptosis in PLD1 deficient mice might be due to reduced intra-organ thrombosis with reduced ischemic events but also a direct effect of PLD1 on cell survival. Pro- and anti-apoptotic properties of PLD1 are described. PLD activity was observed to be a survival signal^50^ and prevents apoptosis in rat fibroblasts and breast cancer cells^51,52^, whereas other studies provide evidence for PLD to be also involved in the induction of cell apoptosis via Src and mTOR signaling^53^ and the p53-dependent cell death pathway^54^. Here we detected reduced cell apoptosis in lung and liver of PLD1 deficient mice that might account for the decreased mortality of PLD1 deficient mice. Recently, platelets were identified to induce cell apoptosis in cancer and neuronal cells^30^. We here detected reduced FasL exposure at the surface of PLD1 deficient platelets (Suppl. Fig. 2A) that unexpectedly did not account for reduced cell apoptosis observed in PLD1 deficient mice because alterations in cell apoptosis were not observed in *Pld1*^*fl/fl*^-PF4 Cre mice with a specific deletion of PLD1 only in platelets.

## Conclusion

Here we identified PLD1 as regulator of TNF-α expression and release upon experimental sepsis. Deficiency of PLD1 protects mice from organ damage, inflammation, massive thrombin generation and cell apoptosis resulting in enhanced survival of mice after LPS induced sepsis. Based on these findings PLD1 is a promising new target to be further developed as a drug candidate for therapy of sepsis.

## Electronic supplementary material

Dataset 1
