# Supplementary material for: Phospholipase D1 regulation of TNF-alpha protects against responses to LPS
Source: Sci Rep. 2018 Jul 3;8:10006. doi: 10.1038/s41598-018-28331-y (PMC6030188; doi:10.1038/s41598-018-28331-y)
Supplement: Supplementary file 1 — Dataset 1 [file 41598_2018_28331_MOESM1_ESM.pdf]

**Phospholipase D1 regulation of TNF-alpha protects against responses to LPS**

**Marc-Andre Urbahn[1*], Sonja Charlotte Kaup[1*], Friedrich Reusswig[1], Irena Krüger[1], Martina Spelleken[1], Kerstin Jurk[2], Meike Klier[1], Philipp A. Lang[3], Margitta Elvers[1*]**

[1]Department of Vascular and Endovascular Surgery, Heinrich-Heine-University University Medical Center, Moorenstraße.5, 40225 Düsseldorf, Germany. [2]Center for Thrombosis and Hemostasis (CTH), University Medical Center Mainz, Germany. [3]Department of Molecular Medicine II, Heinrich Heine University, Düsseldorf, Germany.

## Supplementary Information

## Supplemental Figures

**Figure S1.** Effects of platelet-leukocyte interaction in septic *Pld1*$^{fl/fl}$-PF4 cre + and *Pld1*$^{fl/fl}$-PF4 cre $^{-}$ littermate control mice. **(A)** The number of neutrophils was measured by flow cytometry using Ly-6G antibody. N=6. **(B)** Mac-1 expression at the plasma membrane of neutrophils was measured by flow cytometry. N=6. **(C)** Leukocyte-platelet and (**D**) neutrophil-platelet aggregates at indicated time points. N=7. **(E-F)** Neutrophil recruitment in lungs of PF4-Cre$^{+}$ Pld1$^{fl/fl}$ mice 5h after LPS injection. (**E**) Neutrophils were stained with Ly6G (green). Nuclei were stained with 4’,6’-diamidino-2-phenylindole (DAPI). Merge included staining of platelets with GP9 (red) and is shown in Differential Interference Contrast (DIC) mode. (**F**) Number of neutrophils migrated into lungs was quantified per visual field. N=4, scale bar=50 µm. Data represents the arithmetic mean ± s.e.m

**Figure S2**. Cell apoptosis in *Pld1*$^{fl/fl}$-PF4 cre + and *Pld1*$^{fl/fl}$-PF4 cre $^{-}$ littermate control mice. **(A)** FasL at the platelet membrane was measured in septic and agonist-stimulated platelets. (**B-E**) Lung (**B**) and liver (**D**) sections from healthy (left) and septic (right) PF4-Cre$^{+}$ Pld1$^{fl/fl}$ mice and PF4−Cre$^{-}$ *Pld1*$^{fl/fl}$ littermate controls were stained with active caspase3 antibody (red) and visualized by immunofluorescence microscopy (left panel). Nuclei were stained with DAPI. (**C+E**)The number of caspase-3 positive cells was determined. N=5, scale bar 50 µm. Data represents the arithmetic mean ± s.e.m. (n=3). * $P < 0.05$, ** $P < 0.01$

**Figure S3**. (**A-B**) Lung sections from healthy (left) and septic (right) *Pld1*$^{fl/fl}$-PF4 cre + and *Pld1*$^{fl/fl}$-PF4 cre $^{-}$ littermate control mice were stained with fibrin(ogen) antibody (green), visualized by immunofluorescence microscopy (**A**) and quantified (**B**).

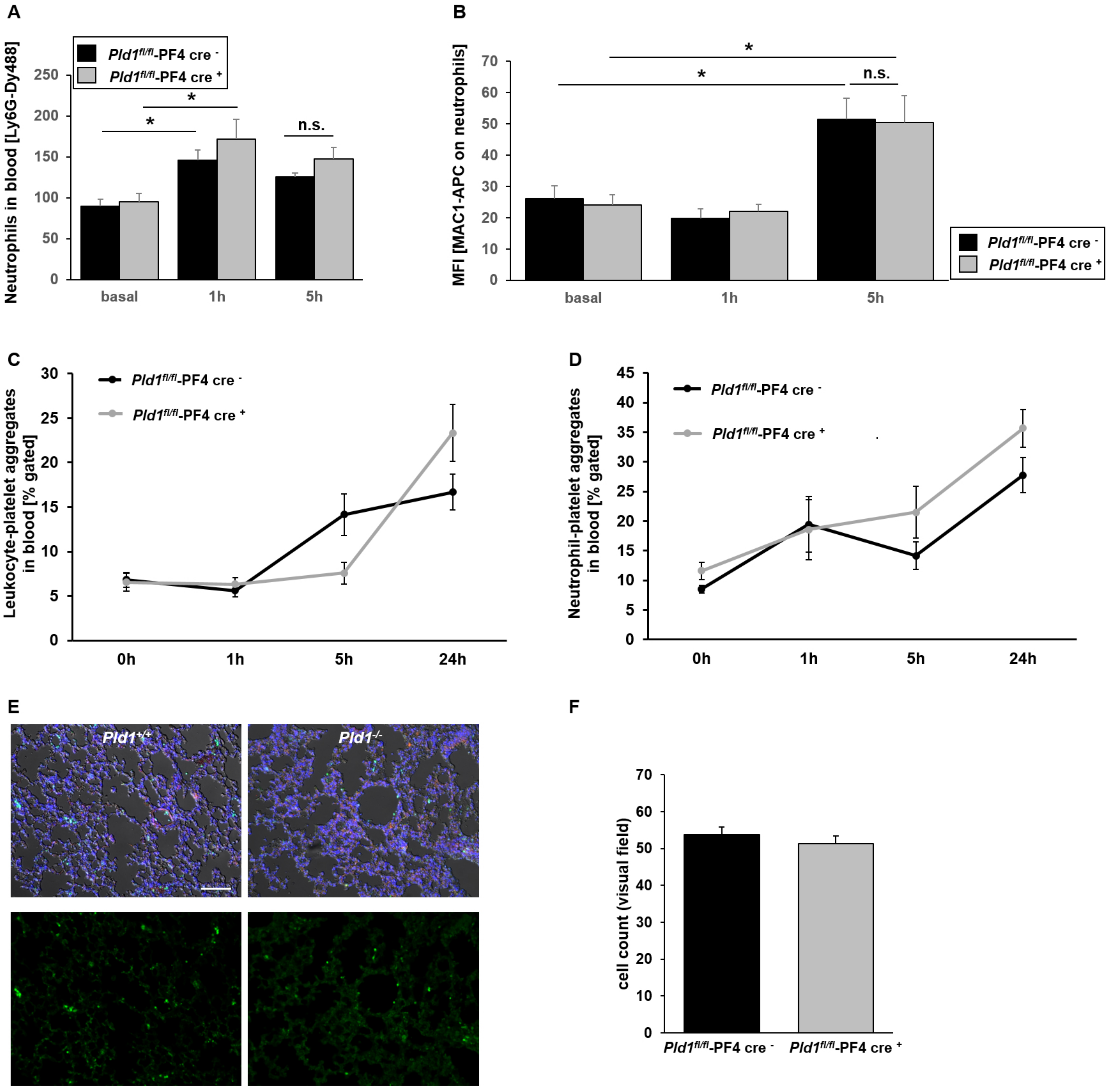
A
Neutrophils in blood [Ly6G-Dy488]
Pld1fl/fl-PF4 cre -
Pld1fl/fl-PF4 cre +
*
*
n.s.
0
50
100
150
200
250
basal
1h
5h
B
MFI [MAC1-APC on neutrophils]
*
*
n.s.
0
10
20
30
40
50
60
70
basal
1h
5h
Pld1fl/fl-PF4 cre -
Pld1fl/fl-PF4 cre +
C
Leukocyte-platelet aggregates in blood [% gated]
Pld1fl/fl-PF4 cre -
Pld1fl/fl-PF4 cre +
0
5
10
15
20
25
30
0h
1h
5h
24h
D
Neutrophil-platelet aggregates in blood [% gated]
Pld1fl/fl-PF4 cre -
Pld1fl/fl-PF4 cre +
0
5
10
15
20
25
30
35
40
45
0h
1h
5h
24h
E
Pld1+/+
Pld1-/-
F
cell count (visual field)
0
10
20
30
40
50
60
70
Pld1fl/fl-PF4 cre -
Pld1fl/fl-PF4 cre +

Suppl.-Fig. 1

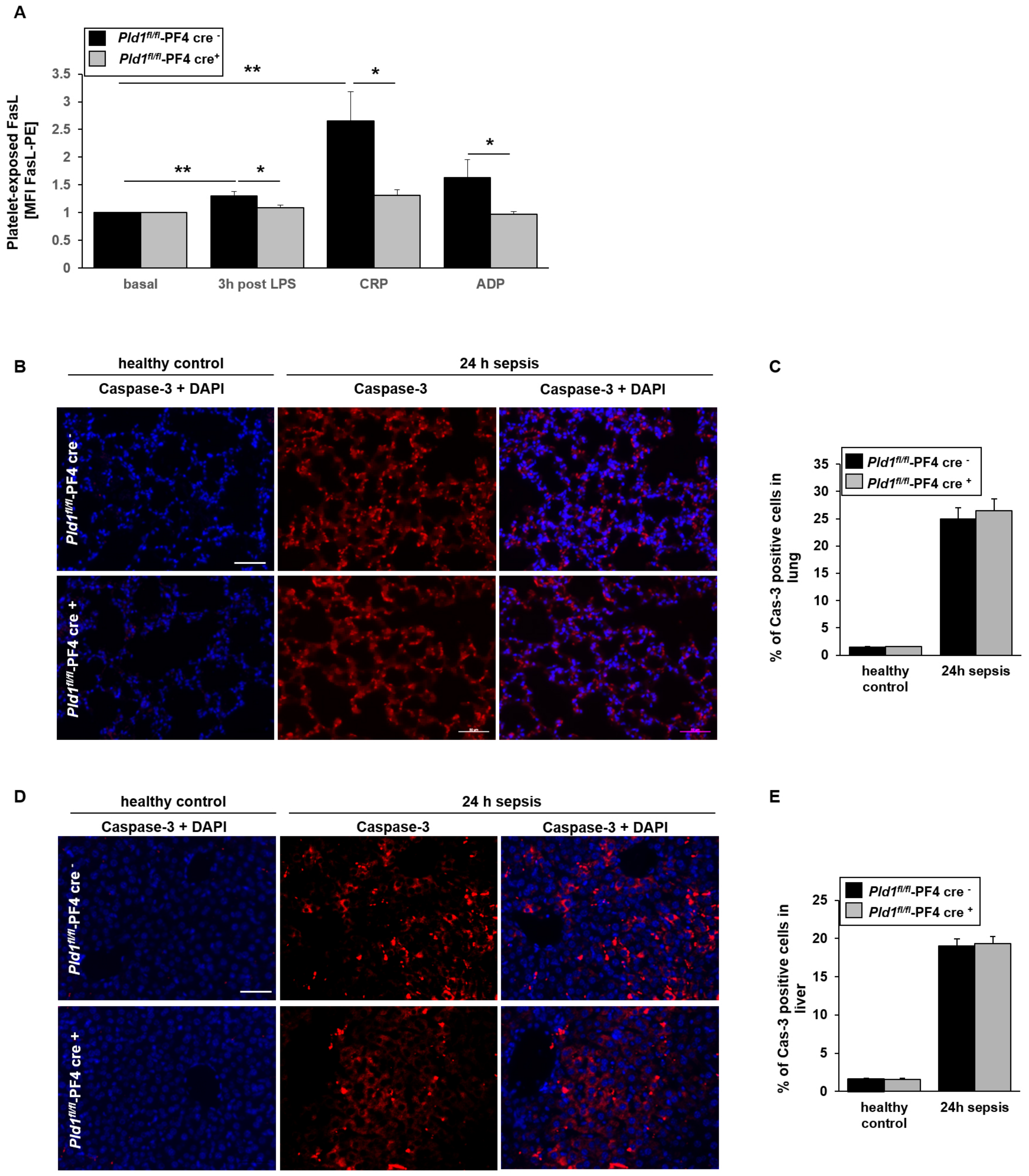
A
Pld1fl/fl-PF4 cre -
Pld1fl/fl-PF4 cre+
Platelet-exposed FasL [MFI FasL-PE]
3.5
3
2.5
2
1.5
1
0.5
0
**
*
**
*
*
basal
3h post LPS
CRP
ADP
B
healthy control
24 h sepsis
Caspase-3 + DAPI
Caspase-3
Caspase-3 + DAPI
Pld1fl/fl-PF4 cre -
Pld1fl/fl-PF4 cre +
C
% of Cas-3 positive cells in lung
35
30
25
20
15
10
5
0
Pld1fl/fl-PF4 cre -
Pld1fl/fl-PF4 cre +
healthy control
24h sepsis
D
healthy control
24 h sepsis
Caspase-3 + DAPI
Caspase-3
Caspase-3 + DAPI
Pld1fl/fl-PF4 cre -
Pld1fl/fl-PF4 cre +
E
% of Cas-3 positive cells in liver
25
20
15
10
5
0
Pld1fl/fl-PF4 cre -
Pld1fl/fl-PF4 cre +
healthy control
24h sepsis

Suppl.-Fig. 2

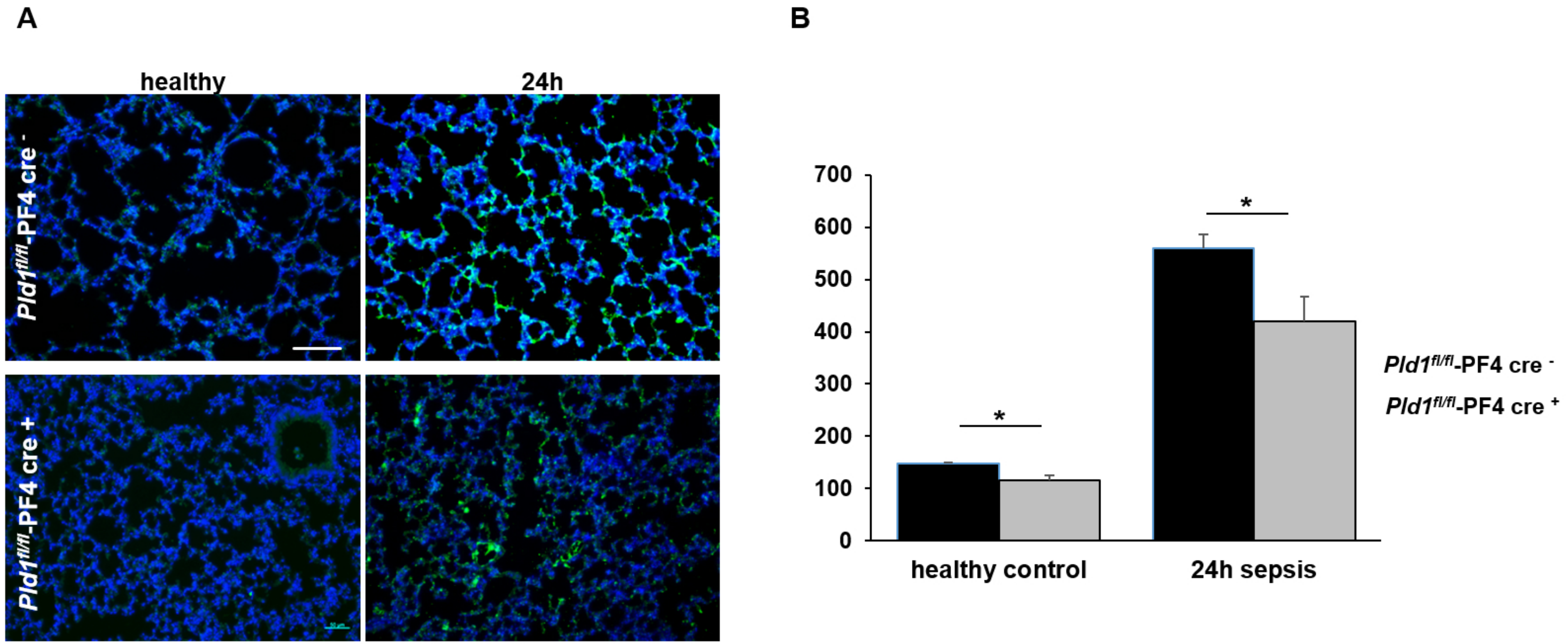
A
healthy
24h
Pld1fl/fl-PF4 cre -
Pld1fl/fl-PF4 cre +
B
700
600
500
400
300
200
100
0
*
*
healthy control
24h sepsis
Pld1fl/fl-PF4 cre -
Pld1fl/fl-PF4 cre +

Suppl.-Fig. 3
